# Receiving antenatal care components and associated factors in Northwestern Tanzania

**DOI:** 10.1371/journal.pone.0284049

**Published:** 2023-04-11

**Authors:** Joseph Massenga, Kidola Jeremiah, Wilson Kitinya, Young-Mi Kim, Jos van Roosmalen, Thomas van den Akker

**Affiliations:** 1 Jhpiego Tanzania, Dar es Salaam, Tanzania; 2 Athena Institute, VU University, Amsterdam, the Netherlands; 3 Mwanza Research Centre, National Institute for Medical Research, Mwanza, Tanzania; 4 Touch Foundation Tanzania, Mwanza, Tanzania; 5 Jhpiego, Baltimore, MD, United States of America; 6 Department of Obstetrics and Gynaecology, Leiden University Medical Centre, Leiden, Zuid-Holland, the Netherlands; University of Gondar College of Medicine and Health Sciences, ETHIOPIA

## Abstract

**Introduction:**

Tanzania had an estimated 5.400 maternal deaths in 2020. Suboptimal quality of antenatal care (ANC) presents a major challenge. It is not known what precisely the uptake of the various ANC components is, such as counseling on birth preparedness and complication readiness, preventive measures and screening tests. We assessed the level of receiving the various ANC components and associated factors in order to identify opportunities to improve ANC.

**Methods:**

A cross-sectional household survey using a structured questionnaire through face-to-face interviews, was conducted in April 2016 in Mara and Kagera regions, Tanzania, applying a two-stage, stratified-cluster sampling design. The analysis included 1,162 women aged 15–49 years who attended ANC during their last pregnancy and had given birth not longer than two years prior to the survey. To account for inter- and intra-cluster variations, we used mixed-effect logistic regression to examine factors associated with receiving essential ANC components: counseling around birth preparedness and complication readiness (with presumed effects on knowledge about danger signs) and preventive measures.

**Results:**

About In 878 (76.1%) women preparedness for birth and its complications was observed to exist. Overall counseling was low where 902 (77.6%) women received adequate counseling. Overall knowledge of danger signs was low in 467 women (40.2%). Uptake of preventive measures was low, with presumptive malaria treatment in 828 (71.3%) and treatment of intestinal worms in 519 (44.7%) women. Screening test levels varied for HIV in 1,057 (91.2%), any blood pressure measurement in 803 (70.4%), syphilis in 367 (32.2%) and tuberculosis in 186 (16.3%) women. After adjusting for age, wealth and parity, the likelihood of receiving adequate counseling on essential topics was less in women without education versus primary education (aOR 0.64; 95% CI 0.42–0.96) and in women who had <4 ANC visits versus ≥4 visits (aOR 0.57; 95% CI 0.40–0.81). Receiving care in privacy or not (aOR 2.01; 95% CI 1.30–3.12) and having secondary education as compared to primary education (aOR 1.92; 95% CI 1.10–3.70) were associated with receiving adequate counseling. Odds of receiving adequate care in at least one ANC visit were lower in women with joint decision making on major purchases versus decision making by male partner or other family members alone (aOR 0.44; 95% CI 0.24–0.78), similar to being less knowledgeable on danger signs (aOR 0.70; 95% CI 0.51–0.96).

**Conclusion:**

Overall uptake of various essential ANC components was low. Frequent ANC visits and ensuring privacy are all essential to improve the uptake of ANC.

## Introduction

Global estimates for 2020 indicated 287. 000 maternal deaths, out of which 70% occurred in sub-Saharan Africa (sSA), the region with the highest Maternal Mortality Ratio (MMR) of 545 per 100. 000 livebirths in 2020 [1]. Tanzania was among the six countries in sSA with more than 5.000 (but fewer than10. 000) maternal deaths in 2020 [1]. The quality of antenatal care (ANC) as part of the whole continuum of care during pregnancy and childbirth is of paramount importance in reducing maternal and perinatal mortality [2, 3]. The World Health Organization (WHO) currently recommends eight ANC-visits to increase opportunities for adequate care and improving pregnancy outcome [4] [NO_PRINTED_FORM].

As Tanzania strives to move a step ahead to reach the status of upper middle-income country from its present lower middle-income status, the health sector is challenged with limited resources to improve quality along the continuum of antenatal, childbirth and postnatal care [5]. Inadequate access to ANC of sufficient quality is one of the major barriers to better pregnancy and childbirth outcomes [6]. Studies have found that insufficient screening for preeclampsia during ANC was associated with increased maternal and perinatal mortality from eclampsia. Less frequent ANC visits than recommended and suboptimal quality of ANC were factors associated with low birthweight [6, 7]. Counseling during ANC is important in improving knowledge and uptake of Reproductive Maternal Newborn, Child and Adolescent health care (RMNCAH). Women who receive counseling during ANC more often make use of skilled birth attendance and family planning (FP) and are reported to have better pregnancy outcomes [8–10].

In Tanzania, only 28.5% of pregnant women use iron-folic acid supplements. At the same time, 28.8% of women aged 15–49 years have anemia, a contributory cause in 14.9% of maternal deaths. Only 56% of pregnant women received two doses of Intermittent Preventive Treatment (IPT) for malaria in 2015, one of the commonest causes of anemia [11–13]. In a rural district in eastern-central Tanzania suboptimal ANC was reported, where hemoglobin was assessed in 22%– 37% and blood pressure in 69% - 87% women during ANC [14].

Inadequate counseling during ANC has been reported in studies from Tanzania, Kenya and Rwanda where only 68% of women were counseled on birth preparedness, 55% on danger signs, 41% on family planning after childbirth and 17% on nutrition [15]. Counseling about danger signs such as fever, vaginal bleeding, swelling of face or legs and convulsions, and instructing women what to do if such signs occur was deficient in many studies from sub-SA [16]. A study in Rwanda reported suboptimal knowledge on danger signs and low birth preparedness among pregnant women attending health facilities [17].

Achieving gender equity and empowering women with regard to decision-making about their own health will make a crucial contribution to progress across all sustainable development goals and targets [9]. Most men have low awareness on pregnancy-related complications, which hampers their participation in maternal health issues [18–23]. Involving men in maternal health care has the potential to improve its uptake [13, 23, 24]. Media exposure is also an important strategy to increase awareness and knowledge in the community and motivate women to attend ANC [18–22].

Kagera and Mara regions in Northwestern Tanzania are among the regions with high numbers of maternal and newborn deaths [25]. Little is known about the level of receiving various ANC components such as counseling on birth preparedness and complication readiness (including danger signs), evidence-based preventive practices and specific factors associated with uptake of these components.

Therefore, aim of this study was to assess uptake of the different components of ANC: preventive practices, counseling on birth preparedness and complication readiness including danger signs, counseling on essential ANC topics and factors associated with receiving these components. Findings are likely to contribute to policy-level strategies and program design in these two regions.

## Methods

### Study design and setting

This was a cross-sectional household survey conducted by the Maternal Child Survival Program (MCSP) in Kagera and Mara regions in Tanzania in April, 2016. MCSP is a global program that was implemented in these two regions, with a focus on improving maternal and newborn health, malaria in pregnancy, postpartum family planning, immunization and pre-service midwifery education.

### Sampling and population

This sub-analysis uses data from 1,263 respondents of reproductive age (15–49 years) who had given birth up to two years prior to the survey. Minimum sample size requirements were calculated under simple random sampling survey design.

**Fig 1:**
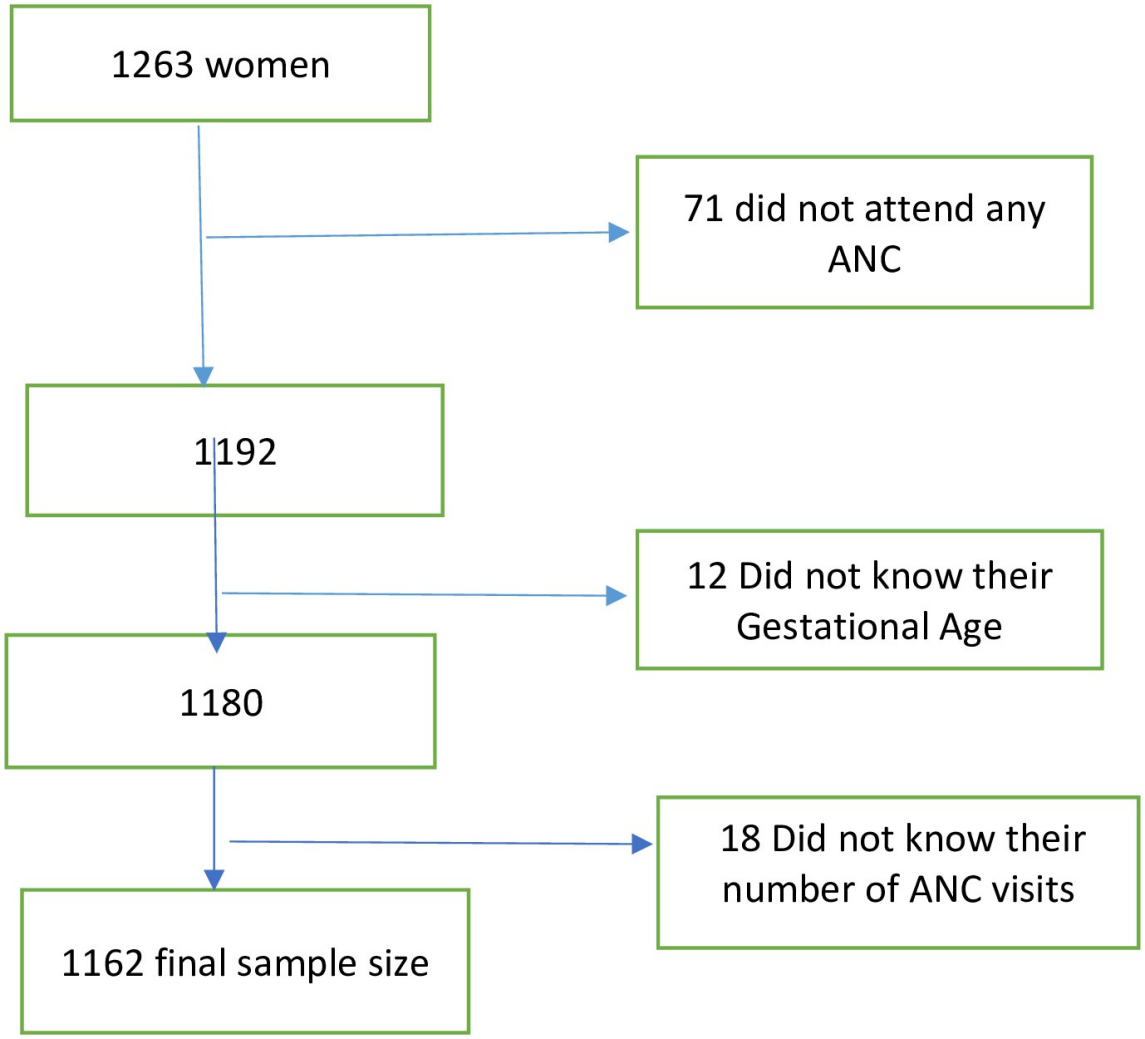
Flow chart showing enrolment of participants.

Women who did not attend ANC (n = 71), those who were unaware of their gestational age (n = 12) and those who did not know their number of ANC visits (n = 18) were excluded. Our study sample thus consisted of 1,162 women who attended at least one ANC visit (Fig 1). Receiving different ANC components and their associated factors were compared between those who received adequate counseling (at least three out of eleven topics) and those with inadequate counseling (less than three out of eleven topics), those who were classified as being knowledgeable on danger signs and those who were not (as defined below) and those who had adequate care in at least one ANC visit (as defined below) or not, with 80% power and level of significance set at 0.05.

We applied a two-stage, stratified-cluster sampling design. Kagera and Mara regions have 333 administrative wards, which were broken down into enumeration areas (EAs). Each EA had approximately 100 households. A total of 32 EAs were selected in each region through the probability-proportional-to-size method. The first household was selected at random by dropping a pen into the generated EA household lists. Additional households were systematically selected from that list until we had interviewed at least 20 women who had recently given birth in one EA. If more than one eligible woman in a household consented to participate, all were interviewed.

Because of cluster sampling, sample size was inflated with a design-effect of 1.5, adjusting for higher intra-cluster correlation. In cases of non-response or non-availability of respondents, no substitutions were made in order to avoid selection bias by the interviewers; therefore, the sample was adjusted by another 10% inflation factor. More details on the study population and sampling technique were previously reported elsewhere [26, 27].

### Operational definitions

#### Outcome variables

**i) Essential counseling topics mentioned during ANC visits**: The first step involved summing up all 11 items, which were reported to be received during ANC counseling as shown in Text Box 1.

**Text box 1.**

Counseling topics which women mentioned to have received information about:

- Danger signs during pregnancy
- Births preparedness and complication readiness
- Nutrition during pregnancy
- Self -care during pregnancy
- Rest during pregnancy
- Danger signs during birth
- Postpartum danger signs for the mother
- Newborn danger signs
- Breast feeding within one hour of birth
- Exclusive breast feeding
- Postpartum family planning

Distribution was symmetrical and this outcome was categorized into those reporting having received at least three out of eleven counseling topics during ANC classified as adequate counseling. This cut-off was based on the Tanzanian context and previously applied in other studies in Haiti and Benin [28, 29].

**ii) Received care in at least one ANC visit**: six care items (HIV testing, blood pressure measurement, urine sample taken, screening for TB, screening for syphilis & blood sample taken). Care content was categorized as ‘adequate’ if all six items had been provided at least once during ANC and was otherwise categorized as ‘inadequate’ [30–33].
**iii) Knowledge of danger signs**: There are three variables regarding knowledge of maternal danger signs -during pregnancy (6 danger signs), childbirth (6 danger signs) and after birth (6 danger signs). In addition, there is a variable on knowledge of newborn danger signs (11 danger sigs). A woman was considered as knowledgeable when she mentioned at least three key danger signs during pregnancy spontaneously. She was considered as knowledgeable when she mentioned at least three key danger signs during childbirth spontaneously. She was considered as knowledgeable when she mentioned at least three key danger signs after childbirth spontaneously. A woman was considered knowledgeable when she mentioned at least three key danger signs for the newborn spontaneously. The overall knowledge on danger signs was derived by dichotomizing the four categories and classify as being knowledgeable (mentioned at least three key danger signs) and less knowledgeable (mentioned less than three key danger signs) [34].

#### Independent variables

We selected independent variables based on the literature that could have an association with outcomes. Exposure to mass media had 19% missing observations and was therefore not included in the regression analysis. Household wealth index was compiled using Principal Component Analysis (PCA) from 18 items present in households (own any livestock, herds, other farm animals or poultry; own any agricultural land; electricity that is connected; radio in working condition; television in working condition; non-mobile telephone in working condition; computer in working condition; refrigerator in working condition; battery or generator for power; watch; mobile phone; bicycle; motorcycle or motor scooter; animal-drawn cart; car or truck; boat with a motor; iron charcoal or electricity; household has bank account). Each item was assigned a factor score and then individual participants were ranked according to the total factor score of the household they belonged to. Wealth index was then developed using PCA into poor, middle and rich [35, 36].

Number of ANC visits was dichotomized as <4 and ≥4 visits. Husband/partner accompanying to ANC was defined as having been accompanied by the husband/partner during at least one counseling visit. Received ANC in privacy: defined as whether a woman felt privacy was present when she was examined, counselled or tested during ANC. ‘Decision making on health’ was a composite variable derived from six elements assessing women’s participation in decision-making related to their own health (I, V and VI) and that of their children (II-IV). These six elements were (I) attending the doctor, (II) accessing child immunizations, (III) child-feeding practices, (IV) health care for sick children, (V) where to give birth and (VI) where to seek care in case of pregnancy complications. Each element had six responses (woman alone; husband alone; woman with her husband jointly; woman and other members in the family jointly; other members in the family alone; decision not made) where in the first step, responses for each element were grouped into three levels: “woman alone”; “jointly” if decision was made together with her male partner or with other members of the family; and “male partner alone or other members in the family”, if women did not participate in decision-making. In the second step, the six elements with the same three levels were then grouped together [37]. A similar approach was used for ‘Decision making on major purchases. Elements included (i) purchase or sell animals, (ii) purchase major goods for the house such as TV, (iii) purchase small household materials such as utensils, (iv) which food to buy for family meals.

*Birth preparedness and complication readiness*. The woman was asked whether during her last pregnancy, she pursued the different elements of birth preparedness, consisting of four elements (save money; arrange transport; decide on birth companion; decide on place of birth). Responses were scored as “0” for no answer and “1” for yes answer. Total score was obtained by computing all elements, which range from 0 to 4 points. Respondents’ scores ≥ 3 were considered as being prepared for birth and <3 as being not prepared [17, 38, 39].

### Data collection

This household survey used structured questionnaires developed by Child Survival Health Grants Program and MCSP [40]. The questionnaire was adapted by reproductive, maternal and child health experts to fit into the local context and then translated into Swahili. Local experts reviewed the Swahili version to ensure its meaning was clear. The questionnaire was pilot-tested by 30 research assistants who were trained on research ethics, study protocol, household sampling, informed consent and other data collection procedures. Research assistants collected data in face-to-face interviews in Swahili for a duration of one hour per participant. Participants’ responses were recorded into password-protected tablets, using CommCare HQ (Dimagi, Cambridge, MA, USA) mobile data collection platform. To ensure data quality, inbuilt skip patterns were included into the questionnaire. On a daily basis, the data manager reviewed the data where study supervisors were immediately alerted on data errors to be addressed.

### Statistical analysis

Data were analyzed in Stata release 14 (Station College, Texas, USA). Descriptive statistical analysis to summarize participant characteristics was performed and presented in both unweighted and weighted percentages. Sampling weights were calculated as N/n, where ‘N’ is the number of elements in the district area and ‘n’ the number of elements in EAs. Since there was no significant difference between unweighted and weighted percentages, results were described using unweighted descriptive statistics.

Differences between categorical variables were compared using Chi-square tests. We investigated factors that influenced outcome variables on the number of counseling topics women reported to have received during ANC, received care in at least one ANC visit and knowledge on danger signs. Since these were nested data from two level-multistage sampling, households were nested within EAs, meaning that each household belonged to only one EA and therefore a two levels mixed-effect logistic regression model was fitted to the data to account for inter- and intra-cluster variations. Accordingly, fixed effects, effects of independent variables in the number of counseling topics, received care in at least one ANC visit and knowledge of danger signs were presented using odds ratios (ORs) with 95% confidence intervals (CIs), while random effects were presented using Intra-Class Correlations (ICCs).

Selection of variables added to the model was based on those previously reported in the literature to be associated with outcome (number of counseling topics women reported to have received during ANC, care provided during ANC and knowledge on danger signs) and those of greater theoretical importance in our study setting. Variables were entered into multiple logistic regression if crude analysis showed p-values < 0.20 [41].

### Ethics approval and consent to participate

This study was approved by the National Research and Ethics Committee (NatREC) in Tanzania with IRB Number NIMR/HQ/R.8a/vol.IX/2131 and the Johns Hopkins Bloomberg School of Public Health Institutional Review Board (IRB Number 5931). All study participants provided oral consent to accommodate various levels of literacy. A research assistant read the consent form aloud in Kiswahili and answered the woman’s questions. When the woman gave her consent, the research assistant wrote down her identification number from a pre-assigned list of identification numbers and signed the form to certify that she gave her consent. For women aged 15–17 years who had a child but were not in union, the consent of a parent or guardian was required in addition to the woman’s own consent for enrolment in the study. The consent procedure and its forms were approved by both IRBs.

## Results

### Participants´ characteristics

The total sample of women aged 15–49 who attended at least one ANC visit in their last pregnancy two years prior the survey was 1,162 (Fig 1) with a median age of 26 years (IQR 21–31). Primary school was completed by 822/1,162 women (70.8%), 257/1,162 (22.1%) were primigravid women and 986/1,123 (87.8%) lived in union. Poor, middle and rich wealth levels were equally distributed, each around 33% (Table 1). Timing of first antenatal visit was often late: 955/1,162 (73.6%) women booked their first ANC visit at a gestational age >3 months and 642/1,162 (55.2%) had attended ≥ 4 ANC visits. Husbands or partners accompanied 673/1,154 (58.3%) women at least once to ANC and 1,017/1,162 (87.5%) women felt privacy was present when they received ANC (Table 1).

**Table 1:** Characteristics of women who had at least one ANC visit in Kagera and Mara regions, Tanzania (n = 1,162).

Characteristics		
	Unweighted	weighted
	n (%)	n (%)
**Mothers age, Median (IQR), years**	26(IQR 21;31)	
**Education level**		
No education	220 (18.9)	220 (19.9)
Primary	822 (70.8)	822 (70.6)
Secondary and above	120 (10.3)	120 (9.5)
**Parity**		
1	257 (22.1)	257 (20.9)
2 to 4	528 (45.4)	528 (44.9)
≥5	377 (32.4)	377 (34.2)
* **Family wealth**		
Poor	377 (33.6)	377 (31.4)
Middle	372 (33.1)	372 (34.1)
Rich	374 (33.3)	374 (34.5)
* **Marriage status**		
In union	986 (87.8)	986 (89.3)
Not in union	137 (12.2)	137 (10.7)
**Gestational age at first ANC**		
≤3 months	307 (26.4)	307 (24.9)
>3 months	855 (73.6)	855 (75.1)
**Number of ANC visits**		
1–3	520 (44.8)	520 (46.6)
4+	642 (55.2)	642 (53.4)
* **Husband/partner accompany to ANC**		
No	481 (41.7)	481 (43.3)
Yes	673 (58.3)	673 (56.7)
**Received ANC in privacy**		
No	145 (12.5)	145 (14.3)
Yes	1,017 (87.5)	1,017 (85.7)
Received counseling topics during ANC		
Inadequate	260 (22.4)	260 (24.4)
Adequate	902 (77.6)	902 (75.6)
* **Received care in at least one ANC visit**		
Adequate	1040 (90.7)	1040 (89.6)
Inadequate	107 (9.3)	107 (10.4)

*Variables with missing values.

ANC = Antenatal care; IQR = Inter Quantile Range.

### Level of receiving ANC components

**i) Knowledge on danger signs.** Of 1,162 women who visited ANC at least once, 467/1,162 (40.2%) were classified as ‘knowledgeable on danger signs’ (Table 2). Among danger signs, vaginal bleeding was the best known at any point during pregnancy (519/1,161; 44.7%), birth (520/1,148; 45.3%) and after birth (714/1,148; 62.2%). Fever was the main danger sign with regard to newborn health (719/1,147; 62.7%). Women’s knowledge of danger signs for newborns was low: only 42/1,132 women (3.7%) mentioned loss of consciousness and 205/1,145 (17.9%) convulsions (Table 2).
**ii) Preventive practices during ANC.** Preparedness for birth was reported in 878/1,154 (76.1%) women and all elements (save money, decision on birth companion and decision on place of birth) were practiced above 80%, except for arranging transport (784/1,160; 67.6%). Decision making on major purchases (676/1,154; 58.6%) and health care (513/1,154; 44.5%) women was often made by either the husband or another family member alone. Taking medicines to prevent intestinal worms (519/1,161; 44.7%) and presumptively treat malaria (828/1,161; 71.3%) as well as sleeping under a mosquito net (1,067/1,162; 91.8%) occurred in different percentages (Table 2).
**iii) Received care in at least one ANC visit.** A total of 1,040 (90.7%) out of 1,147 women who attended at least one ANC visit received adequate care (Table 1). Among women attending ANC, 1,057/1,159(91.2%) were tested for HIV, 803/1,140 (70.4%) had their blood pressure measured at any point during pregnancy, 376/1,138 (32.2%) were screened for syphilis and 186/1,149 (16.2%) for TB (Table 2).
**iv) Received counseling topics during ANC.** Not all topics were covered during ANC counseling. Rest during pregnancy (837/1,162; 72.0%) and exclusive breast feeding (831/1,162; 71.5%) were the most frequently counseled topics and counseling on newborn danger signs occurred at the lowest frequency in 497/1,162 (42.8%) women (Table 3).

**Table 2:** Receiving of ANC components in Mara and Kagera regions, Tanzania.

Characteristics	unweighted	weighted
	n (%)	n (%)
**Women’s knowledge on danger signs during pregnancy**	**1,162**	
Convulsion, Yes	50 (4.3)	50 (3.7)
Headache/Blurred vision, Yes	358 (30.8)	358 (29.8)
Severe Abdominal pain, Yes	375 (32.3)	375 (31.1)
Fever, yes	461 (39.7)	461 (37.6)
Difficulty in breathing, yes	103 (8.9)	103 (7.7)
Vaginal bleeding, yes	519 (44.7)	519 (40.4)
**during childbirth**	**1,149**	
Convulsion, Yes	110 (9.6)	110 (12.2)
High Fever, Yes	222 (19.3)	222 (13.9)
Heavy bleeding, Yes	520 (45.3)	520 (42.7)
Difficulty in breathing, Yes	78 (6.8)	78 (5.8)
Retained placenta, Yes	173 (15.1)	173 (16.2)
Blurred vision, Yes	141 (12.3)	141 (8.1)
**after childbirth**	**1,147**	
Foul-smelling discharge, Yes	127 (11.1)	127 (9.2)
Convulsions, Yes	130 (11.3)	130 (9.9)
Blurred vision/headache, Yes	172 (15.0)	172 (13.6)
High fever, Yes	407 (35.5)	407 (33.0)
Difficulty in breathing, Yes	106 (9.2)	106 (7.7)
Heavy bleeding, Yes *	714 (62.2)	714 (59.9)
**Women’s knowledge on newborn danger signs**	**1,147**	
Baby doesn’t look well, Yes	226 (19.7)	226 (17.1)
Poor suckling or feeding, Yes	438 (38.2)	438 (33.9)
Convulsions, Yes	205 (17.9)	205 (15.7)
Fever, Yes	719 (62.7)	719 (62.2)
Difficulty in breathing, Yes	122 (10.6)	122 (9.4)
Baby feeling cold, Yes	118 (10.3)	118 (10.3)
Baby too small/premature Yes	36 (3.1)	36 (2.6)
Yellow palms/soles/eyes, Yes	91 (7.9)	91 (7.1)
Swollen Abdomen, Yes	89 (7.8)	89 (6.9)
Pus or Redness of umbilical cord., eyes, or skin, Yes	105 (9.2)	105 (8.9)
Unconscious, Yes	42 (3.7)	42 (3.0)
**Overall Knowledge on danger signs**		
Less knowledgeable	695 (59.8)	695 (69.1)
Knowledgeable	467 (40.2)	467 (30.9)
**Women’s practice during ANC:**		
**Birth preparedness/complication readiness**	**1,159**	
Save money, Yes	935 (80.7)	935 (79.0)
Arrange transport, Yes	784 (67.6)	784 (65.9)
Decide on a birth companion, Yes	942 (81.3)	942 (79.7)
Decide on a place of birth, Yes	976 (84.2)	976 (83.2)
**Overall Birth preparedness/ complication readiness**		
Not prepared	276 (23.9)	276 (25.6)
Prepared	878 (76.1)	878 (74.4)
**Decision making on health care**	**1,154**	
Woman Alone	359 (31.1)	359 (30.0)
Husband/others alone	513 (44.5)	513 (48.2)
Jointly with man/others	282 (24.4)	282 (22.8)
**Decision making on major purchases**	**1,154**	
Woman Alone	69 (6.0)	69 (5.8)
Husband/others alone	676 (58.6)	676 (61.1)
Jointly with man/others	409 (35.4)	409 (33.1)
**Taking drugs for intestinal worms, Yes**	519 (44.7)	519 (44.2)
**Taking medicine to prevent malaria, Yes**	828 (71.3)	828 (67.7)
**Sleeping under mosquito net during pregnancy, Yes**	1,067 (91.8)	1,067 (94.0)
**Accompanied with husbands during ANC visits, Yes**	673(58.3)	673(56.7)
**Screening tests**		
Tested for HIV, Yes (n = 1,159)	1,057 (91.2)	1,057 (89.4)
Blood pressure measured (n = 1,140)	803 (70.4)	803 (70.1)
Urine sample taken (n = 1,150)	557 (48.4)	557 (46.6)
Screening for TB (n = 1,149)	186 (16.3)	186 (15.2)
Screened for Syphilis (n = 1,138)	367 (32.2)	367 (29.9)
Blood sample taken (n = 1,156)	1,033 (89.4)	1,033 (88.1)

The percentages were calculated by taking number of observations ‘‘Yes” out of total observations for each variable.

**Table 3:** Proportion of women who reported received counseling on various topics during ANC in Mara and Kagera, Tanzania (n = 1,162).

	Unweighted			Weighted		
Topic	Yes	No	Don’t know	Yes	No	Don’t know
	n (%)	n (%)	n (%)	n (%)	n (%)	n (%)
Danger signs during pregnancy	639 (55.0)	503 (43.3)	20 (1.7)	639 (68.7)	503 (30.5)	20 (8.6)
Birth preparedness	765 (65.8)	373 (32.1)	24 (2.1)	765 (66.0)	373 (32.7)	24 (1.3)
Nutrition during pregnancy	744 (64.0)	406 (35.0)	12 (1.0)	744 (63.8)	406 (34.1)	12 (2.1)
Self-care during pregnancy	794 (68.3)	353 (30.4)	15 (1.3)	794 (61.8)	353 (37.2)	15 (1.0)
Rest during pregnancy	837 (72.0)	319 (27.5)	6 (0.5)	837 (60.0)	319 (38.7)	6 (1.3)
Danger signs during birth	519 (44.7)	616 (53.0)	27 (2.3)	519 (29.7)	616 (69.7)	27 (5.6)
Postpartum danger signs for the mother	562 (48.4)	578 (49.7)	22 (1.9)	562 (44.5)	578 (53.8)	22 (1.7)
Newborn Danger signs	497 (42.8)	641 (55.2)	24 (2.0)	497 (42.2)	641 (55.5)	24 (2.3)
Breast feeding within one hour of birth	736 (63.3)	409 (35.2)	17 (1.5)	736 (57.3)	409 (40.8)	17 (1.9)
Exclusive breast feeding	831 (71.5)	321 (27.6)	10 (0.9)	831 (52.1)	321 (46.2)	10 (1.7)
Postpartum family planning	757 (65.2)	401 (34.5)	4 (0.3)	757 (64.9)	401 (34.7)	4 (0.4)

### Factors associated with receiving ANC components: Number of counseling topics, received care in at least one ANC visit and women’s knowledge on danger signs

**i) Received counseling topics during ANC.** The proportion of women who received adequate counseling on essential topics during ANC was 902/1,162 (77.6%). Women without education had lower odds of receiving adequate counseling than those with primary education (aOR 0.64; 95% CI 0.42–0.96). Women with secondary education and higher education had two times higher odds of receiving adequate counseling than those with primary education (aOR 1.92; 95% CI 1.10–3.70). Women in Kagera region reported twice as higher odds to have had adequate a counseling than those from Mara region (aOR 2.39; 95% CI 1.45–3.95). Women who made <4 ANC visits had lower odds of receiving adequate counseling (aOR 0.57; 95% CI 0.40–0.81) than those who made ≥4 ANC visits. Women who felt their privacy existed during care, reported twice as higher odds to receive adequate counseling compared to those who experienced lack of privacy (aOR 2.01; 95% CI 1.30–3.12) (Table 4).
**ii) Received care in at least one ANC visit.** About 1,040/1,147 (90.7%) women received adequate care. Women who made <4 ANC visits had lower odds of receiving adequate care than those who made ≥4 ANC visits (aOR 0.58; 95% CI 0.35–0.96). Odds of receiving adequate care were lower in women who reported joint decision making on major purchases compared to those who reported decisions were made by the husband/partner alone or other family members alone (aOR 0.44; 95% CI 0.24–0.78) (Table 4).
**iii) Knowledge on danger signs.** About 467/1,162 (40.2%) women were classified as being knowledgeable on danger signs. Women who reported joint decision making on major purchases had lower odds of knowledge of danger signs compared to those who reported decisions are made by husband/partner alone/other members in the family alone (aOR 0.70; 95% CI 0.51–0.96) (Table 4). A substantial number of women (673/1,154; 58.3%) reported that their husbands had accompanied them at any point during ANC. This was, however, not associated with having received counseling of more than three topics, receiving adequate ANC or having adequate knowledge about danger signs (Table 4).

**Table 4:** Mixed effect logistic regression analysis examining factors associated with receiving ANC components (number of counseling topics, received care in at least one ANC visit, and women’s knowledge on danger signs) in Mara & Kagera*regions.

Variable	Received counseling topics during ANC	Received care in at least one ANC visit	Knowledge on danger signs
	Inadequate vs. Adequate	Adequate care vs inadequate care	Less knowledgeable vs knowledgeable
	N = 1,116	N = 1,103	N = 1,116
	aOR (95% CI)	aOR (95% CI)	aOR (95% CI)
**Age category**			
15–24	0.97 (0.62–1.51)	0.82 (0.44–1.55)	1.16 (0.81–1.66)
25–34	Ref		
35 and above	1.40 (0.83–2.38)	0.80 (0.38–1.69)	1.32 (0.87–2.01)
**Education level**			
No Education	0.64 (0.42–0.96)	0.85 (0.43–1.67)	0.75 (0.52–1.09)
Primary	Ref		
Secondary and above	1.92 (1.10–3.70)	0.83 (0.40–1.73)	1.37 (0.87–2.16)
**Wealth category**			
Middle	Ref		
Poor	0.90 (0.59–1.37)	1.59 (0.93–2.72)	1.28 (0.91–1.78)
Rich	0.83 (0.55–1.25)	0.62 (0.33–1.19)	0.80 (0.57–1.13)
**Region**			
Mara	Ref		
Kagera	2.39 (1.45–3.95)	1.72 (0.78–3.37)	1.19 (0.77–1.86)
**Parity**			
1	0.67 (0.41–1.09)	1.44 (0.74–2.80)	0.70 (0.47–1.03)
2 to 4	Ref		
5+	0.99 (0.64–1.54)	1.23 (0.66–2.30)	1.09 (0.76–1.56)
**Marriage status**			
In union	Ref		
Not in union	1.22 (0.68–2.18)	1.06 (0.46–2.44)	0.98 (0.60–1.58)
Number of ANC visits			
4+ visits	Ref		
1 to 3 visits	0.57 (0.40–0.81)	0.58 (0.35–0.96)	0.81 (0.61–1.08)
**Decision making on purchasing major goods**			
Man/other alone	Ref		
Woman alone	1.04 (0.48–2.26)	1.04 (0.38–2.88)	0.75 (0.40–1.41)
Jointly	0.80 (0.56–1.16)	0.44 (0.24–0.78)	0.70 (0.51–0.96)
**Decision making on health care**			
Man/other alone	Ref		
Woman alone	1.27 (0.85–1.92)	0.81 (0.46–1.42)	0.93 (0.66–1.30)
Jointly	1.20 (0.79–1.84)	0.65 (0.34–1.22)	1.25 (0.88–1.76)
**Husband/partner accompany to ANC**			
No	Ref		
Yes	1.36 (0.96–1.94)	1.10 (0.65–1.85)	0.97 (0.72–1.31)
**Felt privacy existed during care**			
No	Ref		
Yes	2.01 (1.30–3.12)	1.23 (0.55–2.76)	1.35 (0.88–2.06)
**GA at first ANC**			
>3months	Ref		
≤3 months	0.90 (0.60–1.35)	0.95 (0.56–1.60)	0.87 (0.63–1.20)
**Random- effects**			
ICC	0.13 (0.07–0.25)	0.19 (0.10–0.34)	0.13 (0.07–0.21)

*Total observations do not add up to 1,162 because some variables had missing observation in the regression analysis.

## Discussion

Uptake of the various ANC components was low. Less than two thirds of the women had were classified as being knowledgeable about danger signs. Preventive practices occurred in varying proportions. More than three out of four women were classified as being prepared for birth, while the use of medications to prevent intestinal worms and malaria and the decision-making power of women were low. Less than three-quarters mentioned to have received adequate counseling on essential topics and counseling on newborn danger signs was particularly low.

Having completed secondary education or higher, having ≥4 ANC visits and having felt that privacy existed during care were associated with adequate counseling on essential topics. Having ≥4 ANC visits and decision making by the husband alone or other members in the family alone were associated with receiving adequate care during ANC. Decision making by the husband alone or other members in the family alone was associated with more knowledge on danger signs.

As found in studies from Nigeria and Ghana, our findings showed that women who felt privacy during ANC have higher likelihood of receiving more counseling topics [42, 43]. Women felt more comfortable attending counseling sessions when privacy is guaranteed, likely because they feel free to share information [44]. Lack of privacy has been reported as a key barrier to accessing and receiving quality ANC [45–48]. Ensuring privacy reduces fear and offers opportunities to discuss a broader range of health issues [42, 43, 49].

Large numbers of women reported that their husbands accompanied them during at least one ANC visit. This was, however, not statistically significantly associated with more topics mentioned during ANC counseling or with getting adequate care or obtaining more knowledge about danger signs. This may be explained by the fact that in Tanzania, men who accompany their partners to health facilities for maternity care, remain outside the facility during ANC or during labour and birth while their partners receive care inside [50, 51]. Inadequate infrastructure or provider’s attitudes may not allow couples to be together during counselling in ANC or childbirth [11, 52, 53]. There is a need for partner-friendly policies that address provider’s attitudes and infrastructure in the various ANC visit points that would make men and women more comfortable to accompany their partners [54].

In contrast with other studies our findings showed that decision making by the husband alone or other members in the family alone compared to joint decision making was associated with receiving adequate care and knowledge of danger signs [55–57]. Our findings are similar to those in Senegal, where women who make decisions on major household purchase solely or jointly with their husbands had lower odds of utilizing skilled birth attendance [55] and in Nigeria where women’s decision autonomy reduced likelihood of early initiation of ANC [37]. Our findings can be explained by the fact that male dominance in Tanzania is still impacting the extent to which women actually receive RMNCAH care as well as their knowledge of danger signs. According to the Tanzanian DHS 2015/2016, male dominance exists more frequently in Mara and Kagera regions as compared to other regions due to social norms in these regions [25]. Programs focusing on improving the continuum of maternity care in these regions need to challenge male dominance and promote women’s decision-making power and male awareness of its importance [56, 57].

Women who made 4+ ANC visits as compared to those who made fewer received more counselling topics and adequate care, which is consistent with studies in Haiti, Ethiopia and Kenya [29, 58–60]. and current WHO guideline recommends 8 number of ANC contacts/visits [4]. More frequent ANC visits increase opportunities to interact with health care workers and potentially enhance health talks and ANC in general [61, 62]. Promoting the number of ANC visits should go together with investing in human and material resources to ensure better quality ANC [14].

### Strengths and limitations

The use of a stratified cluster sampling technique, which accounts for between and within cluster variations and the large sample size are strengths of this study. No causal inferences, however, can be established from a cross-sectional method. The sample was drawn from only two regions out of twenty-six in Tanzania, limiting generalizability of the findings. We were not able to report any information on determinants of ANC from seventy-one women who did not attend any ANC.

## Conclusion

Overall, uptake of the different ANC components was low Ensuring privacy and frequent ANC attendance, and improving quality of counseling are all needed to improve ANC. Male involvement should go beyond escorting partners to clinics and involve their actual presence during provision of care to improve ANC components. Programs designed to improve ANC should include women’s empowerment and strengthen the entire continuum of care.
